# Recovery of pure PET from wool/PET/elastane textile waste through step-wise enzymatic and chemical processing

**DOI:** 10.1177/0734242X241276089

**Published:** 2024-09-20

**Authors:** Emanuel Boschmeier, Daniella Mehanni, Viktor Laurin Sedlmayr, Yury Vetyukov, Sophia Mihalyi, Felice Quartinello, Georg M Guebitz, Andreas Bartl

**Affiliations:** 1 Institute of Chemical, Environmental and Bioscience Engineering, TU Wien, Vienna, Austria; 2 Institute of Mechanics and Mechatronics, TU Wien, Vienna, Austria; 3 Department of Agrobiotechnology, IFA-Tulln, Institute of Environmental Biotechnology, University of Natural Resources and Life Sciences, Tulln an der Donau, Austria

**Keywords:** Textile recycling, enzymes, wool removal, elastane separation, PET recovery, keratin hydrolysate

## Abstract

Textile waste is mostly incinerated because few recycling processes are available to recover valuable materials. In this work, a feasible chemo-enzymatic recycling process of wool/polyethylene terephthalate (PET)/elastane blends to recover pure PET is for the first time successfully demonstrated. Two novel enzyme formulations were selected for wool hydrolysis, whereas the recovered amino acids were quantified using high-performance liquid chromatography and two assays (Ninhydrin and Folin–Ciocalteu). Kinetic studies on the amino acid formation alongside reaction observations by scanning electron microscopy proved sufficient removal of wool within 8 hours with the new enzyme formulation, marking an acceleration compared to previous studies. Finally, elastane was separated with a non-hazardous solvent to obtain pure PET. Tensile tests on the recovered PET fibres reveal only slight changes through the enzymatic treatment and no changes induced by the applied solvent. The enzyme formulation was successfully tested on five different post-consumer wool/PET textile waste samples. This valorization approach enhances the circular economy concept for textile waste recycling.

## Graphical abstract

**Graphical Abstract:**
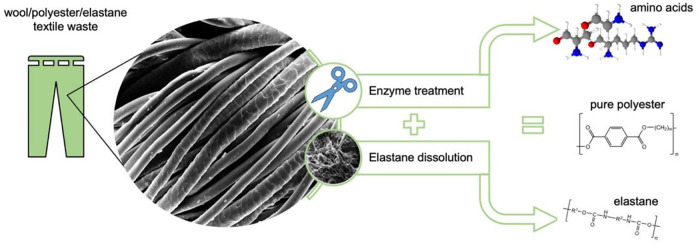
This is a visual representation of the abstract.

## Highlights

- First combination of two different non-hazardous recycling methods
- Accelerated wool removal by application of a proteolytic enzyme formulation
- Recovery of pure PET fibres after full separation of elastane
- Successful field test with the novel enzyme formulation

## Introduction

Textiles represent an essential product group that the growing population needs every day. Therefore, fibre production was rising from 34 Mio t in 1975 to 113 Mio t by 2021 and is expected to grow further to 149 Mio t by 2030 (Textile Exchange, 2022). Compared to other waste streams, such as packaging, the management of textiles is still underdeveloped. In the European Union (EU), 78% of textile waste, in total 12.6 Mio t by 2019, was disposed together with residual waste and thus thermally recovered or landfilled. Even the 22% of textiles collected separately are only partially reused or recycled in the EU, with a large proportion going for export (EC, 2022) into low-income countries (Boschmeier et al., 2024). Next to the loss of potential secondary raw material, such exported waste stream is frequently not sustainably handled due to lower environmental standards in waste management (Poon et al., 2024). Due to upcoming legal requirements for EU member states in the next years, such as mandatory separate collection of textile waste (EC, 2018), an extended producer responsibility (EPR) system (EC, 2023) and incentives on more sustainable, repairable and circular products (EC, 2024), reuse and in particular recycling systems must be massively enhanced. The textile and waste management sector are therefore urgently seeking for recycling solutions for textile waste. However, textile waste recycling is rather complex due to the multi-material compositions, accessories (buttons, zippers) and post-consumer feedstock condition (Boschmeier et al., 2024). Furthermore, due to the long production chain, there are different entry points into the textile recycling processing chain for closing the loop. In particular, it is possible to feedback material on the fibre, polymer or monomer level (Ipsmiller and Bartl, 2022). It is common practice to produce textiles containing different kind of fibre materials. Blending natural fibres (e.g. wool) with synthetic fibres such as polyethylene terephthalate (PET) or elastane (EL) results in beneficial properties whilst keeping production costs at a minimum (Lebedytė and Sun, 2022).

Wool fibres in textiles improve wear comfort and insulative properties as well as ameliorated odour-resistance (Wiedemann et al., 2020) and are animal-based fibres mainly retrieved from sheep. Approximately 95–98% of wool is attributed to the main component keratin, a protein fibre also found in human hair that characteristically exhibits a coiled-coil structure. This structure consists of strong oriented cortical cells that contain the keratin, a protein consisting of different amino acids that are bonded together by disulphide bridges (Giteru et al., 2023). In 2021, the wool production was 1.0 Mio t, which is just 0.92% of the total fibre production (Textile Exchange, 2022). Today, open-loop and closed-loop recycling processes are available for wool on an industrial scale, where for closed-loop cycles the wool content should preferably be above 80% (Russell et al., 2016). Importantly, wool tends to felt, especially in machine washing (El-Sayed, 2022) which can be difficult for existing wool recycling lines (Bianco et al., 2022; Testa et al., 2017). By adding PET fibres to textiles, the shrinking resistance and water repellence amongst other properties can be improved (Deopura et al., 2008), but most critical, material costs are lowered. Compared to wool fibres, PET fibres are much cheaper procurable on the highly competitive textile brand market (Boschmeier et al., 2024) and by far the most important fibre, contributing to 64% of global fibre production in 2021, with a production volume of 60.5 Mio t (Textile Exchange, 2022).

EL fibres, based on a segmented polyurethane (Boschmeier et al., 2023a), are usually incorporated in textiles in low amounts and enhance the textiles stretchability. Despite the low production quantities of elastane, 1.2 Mio t in 2021 (Textile Exchange, 2022), this highly elastic fibre is present in many textiles due to its beneficial properties. Even if apparel containing elastane exhibits superior wear-comfort, the presence of elastane significantly hinders any recycling process of blended textiles (Boschmeier et al., 2023b). A mandatory first step in textile recycling is the separation of multi-material fabrics to obtain only pure components for revalorization.

One promising separation technique is the approach of using enzyme cocktails to remove natural fibres. In the case of wool, like in nature, enzymes can degrade the protein fibres under mild processing conditions into their monomers, amino acids, whereas other components of blended textiles such as PET fibres remain intact. A wool hydrolysate enriched with different amino acids is obtained that can be applied in industry applications like fertilizer production (Fang et al., 2013; Li, 2022) and additive in cosmetic products (Mokrejs et al., 2011; Villa et al., 2013). Enzymatic approaches for recycling of textiles containing wool fibres have been previously studied (Eslahi et al., 2013; Navone et al., 2020; Quartinello et al., 2018). However, additionally removal of elastane was not considered so far.

This paper presents the first combination of a chemo-enzymatic recycling process for wool/PET/EL textile blends. In principle, two ways for separation of these blends would be possible. On the one hand, PET could first be dissolved by chemical (Lang et al., 2023), biochemical (Sonnendecker et al., 2022; Tournier et al., 2020) or synergistic chemical/enzymatic approach methods (Quartinello et al., 2017) and on the other hand, one could first remove the wool share. In both cases, a blend of two fibres will be obtained, which requires subsequent removal of elastane. In this work, the wool was separated by enzymatic hydrolysis in a first step since recycling of wool fibres is rather difficult due to their damage during use. Fibre length, a crucial parameter for textile quality, is reduced already during the use-phase of textiles (Aronsson and Persson, 2020) as well as during the tearing process itself (Lindström et al., 2020). On the other hand, it is known from literature that EL can be separated from blends with PET and PA (polyamide) (Boschmeier et al., 2023b), but there is no information available on how to remove EL from blends with wool. Another advantage of PET over wool fibre recycling is that the recovered PET can be used for a new fibre drawing process (Gritsch et al., 2023; Piribauer et al., 2021).

## Materials and methods

### Textile waste samples

A beige-coloured pre-consumer textile waste sample supplied by a Belgian fashion brand with the labelled composition of 53% PET, 44% wool and 3% elastane (wool/PET/EL) was the test substrate for enzyme investigations. The best-performing enzyme was tested then on five pre-sorted wool/PET post-consumer textile waste samples, which come from a textile waste sorting organization. These grey and black coloured samples contained unknown shares of wool and PET. The composition was retrieved from the mass difference before and after enzymatic treatment. PET reference fabric was procured from Komolka KG (Vienna, Austria). All samples were used as delivered without any pre-washing.

### Enzymes, characterization and treatment of wool fibres

From previous literature (Eslahi et al., 2013; Quartinello et al., 2018) the well-known enzyme formulation for the selective hydrolysis of wool fibres Savinase 12T^®^ (Savinase) was used. Next to that, two novel enzyme cocktails namely NS 59161 (enzyme A, density 1.16 g mL^−1^) and NS 29083 (enzyme B, density 1.08 g mL^−1^) were kindly provided by Novozymes (Copenhagen, Denmark). Both are new developed commercially available enzymes and are tested in this work on their performance in wool fibre degradation. All enzyme formulations were used as delivered and can be described as subtilisin proteases with optimal reaction conditions in alkaline conditions.

Enzyme activity determination was carried out using an Azocasein-assay protocol by a serial dilution of different substrate concentrations (1:1, 0.08–20 g L^−1^). In this protease activity assay, 75 µL of enzyme was added to 125 µL of Azocasein solution. After 30 minutes of incubation at 37°C, 600 µL of trichloroacetic acid (10%; Sigma Aldrich, Vienna, Austria) was added to terminate the reaction. Pure buffer without the enzyme was used for the blank determination. The samples were incubated for 15 minutes at 25°C and centrifuged at 13,000 rpm. Subsequently, 600 µL of the supernatant was mixed with 700 µL of 1 M sodium hydroxide solution. Absorption was measured at 440 nm using a Spark^®^ spectrophotometer (TECAN, Männedorf, Switzerland) in triplicates.

Prior to the enzymatic treatment, the wool/PET/EL samples were cut into approximately 6 × 6 mm pieces. In order to have identical experimental conditions, the cut-outs were pre-dried at 60°C for 12 hours. For yield determination during the enzymatic treatment, 3 g of the textile sample were incubated at 50°C in 100 mL 50 mM TRIS–HCl buffer pH 9 in presence of 6 g L^−1^ sodium bisulphite (Sigma Aldrich) and 1 g L^−1^ sodium dodecyl sulphate (SDS; Sigma Aldrich) for 48 hours using 2.5 vol.-% of enzyme A and B. The reaction was stopped by adding 2.5 mL of pure methanol and the keratin hydrolysate was stored at 4°C. The treated samples were washed in 100 mL mQ-H_2_O and dried at 60°C for 12 hours. Experiments were conducted in duplicates. For yield determination and kinetic studies of the enzymes A and B, samples were taken after different treatment duration: 1–8, 12 and 48 hours.

The mass yields were calculated using the quotient of the mass difference of the untreated (*m*_2_) and treated (*m*_1_) fabrics while including the share of wool present in the garment (factor *f*) according to equation (1):

(1) $$\mathrm{mass}\;\mathrm{yield}\;\left(\%\right)=\frac{m_{2}-m_{1}}{f*m_{2}}*100$$

The removal of woollen fibrous residues to guarantee fabrics purity was achieved through extensive studies on post-treatment methods. The following solvents were used for the removal of the fibrous leftovers: ethanol, dimethyl sulphoxide (DMSO) and water (see Supplemental Table S1).

For larger scale wool removal of post-consumer textile waste, an incubator with a reaction volume of 4 L was used and the reagent ratios were maintained as following: 2.5 vol.-% enzyme, 1 wt.-% SDS and 6 wt.-% sodium bisulphite. Two hundred grams of sample were treated for 48 hours and later washed in 100 mL mQ-H_2_O.

### Ninhydrin assay and Folin–Ciocalteu assay

The concentration of soluble amine groups in the wool hydrolysate released by enzymatic treatment was assessed via the Ninhydrin assay. For calibration, glycine standards in the range of 0–200 µM were prepared. Seventy-five microlitres of the ninhydrin reagent was added to 100 µL sample/standard and incubated for 30 minutes at 80°C. Then, the samples were cooled to room temperature. Finally, 100 µL of 50% ethanol was added as a stabilizing solution. The absorbance was measured in duplicates at 570 nm in an Infinite 200 Pro spectrophotometer (TECAN).

Phenolic compounds content determination was assessed by the Folin–Ciocalteu assay. Standard samples (1–0.05 g L^−1^) were prepared using a vanillin stock solution (1 g L^−1^). Sixty microlitres of Folin–Ciocalteu reagent, 20 µL of sample or standard solution and 600 µL of mQ-H_2_O were vortexed and incubated at room temperature for 5–8 minutes. Subsequently, 200 µL of a 20% sodium carbonate solution and 120 µL of mQ-H_2_O were added. The samples were then vortexed and shaken for 2 hours at 21°C and 800 rpm. Absorbance was measured at 760 nm in an Infinite 200 Pro spectrophotometer (TECAN). In parallel, blanks for the enzymes and samples were prepared which were subtracted from the absorbance, respectively.

### Analysis of amino acids via high-performance liquid chromatography

High-performance liquid chromatography (HPLC) analysis of amino acids was carried out on an Ultimate 3000 (Thermo Scientific, Waltham, MA, USA) machine equipped with a reversed phase column (150 × 3 mm, 3.5 µm; Agilent ZORBAX Eclipse AAA (Agilent, Vienna, Austria)) and a precolumn (12.5 × 3 mm, 5 µm; Agilent Eclipse AAA). The mobile phase was composed of 40 mM NaH_2_PO_4_ × H_2_O, pH = 7.8) and eluent B (methanol:acetonitrile:mq = 45:45:10). The flow rate and the column temperature were 1.2 mL min^−1^ and 40°C, respectively. After 2.5 minutes of equilibration at 100% A, a gradient was performed for 17.5 minutes to 48.5% buffer B. At 20.5 minutes, a 3-minute-long washing step was conducted, followed by a re-equilibration step for 2.5 minutes. For detection via fluorescence, an in-needle pre-column derivatization step was performed. For primary amino acids, ortho-phthaldialdehyde containing 1% 3-mercaptopropionic acid and for secondary amines fluormethylencarbonylchloride was used as a derivatization agent. Norvaline and sarcosine served as internal standards and were added with a final concentration of 1.25 mM to all samples and standards. Primary amines and norvaline were detected at 340/450 nm (excitation/emission) and secondary amines and sarcosine were detected at 266/305 nm (excitation/emission).

### SDS–poly acrylamide gel electrophoresis

SDS–poly acrylamide gel electrophoresis (SDS-PAGE) was performed to qualitatively determine the molecular mass distribution of poly- and oligopeptides released during the hydrolysis process. As a reference hydrolysis sample, keratin was extracted from 1 g of untreated textile sample in 10 mL of 50 mM TRIS–HCl pH 8.5 supplemented with 1.5 M 2-mercaptoethanol, 8 M urea and 0.25 M SDS for 4 hours at 60°C. Twenty microlitres of samples during the hydrolysis process of enzyme A (1, 2, 8, 16 and 48 hours) and enzyme B (8, 16 and 48 hours) and the reference hydrolysis sample were mixed with 20 µL of 2× Laemmli buffer. After incubation at 95°C for 5 minutes, 20 µL of the mixtures were transferred on a precast polyacrylamide gel (4–15%; Mini-PROTEAN^®^ TGX (Bio-Rad, Feldkirchen, Germany)). Seven microlitres of Precision Plus Protein Unstained Standards (10–250 kDa) was used as a protein ladder. After development at 180 V, the gel was stained in staining solution (0.05 g mL^−1^ Coomassie Brilliant Blue in 60% mq, 30% methanol, 10% acetic acid) and destained in 60% mq, 30% methanol and 10% acetic acid. Imaging was performed on a Molecular Imager Gel Doc XR System (Bio-Rad, Feldkirchen, Germany).

### Kinetic investigation of wool hydrolysis

One-phase exponential decomposition function with time constant parameter (equation (2)) was chosen to mathematically describe the affinity of the novel enzymes on the substrate and to identify the best performer. Herein, for each enzyme the reaction evolution was retrieved from the released amine groups via Ninhydrin assay. Values for initial quantity *A*, rate constant λ and offset *A*_0_ were obtained from the results at the sample taking points *t* and non-linear curve fitting was calculated with Levenberg–Marquardt iteration algorithm in Origin Pro (OriginLab, Northampton, MA, USA). The necessary time duration to reach 50% conversion rate *t*_1/2_ is taken as a performance indicator and is calculated by equation (3).

(2) $$\mathrm{Concentration}\left(\frac{\mathrm{mmol}}{L}\right)=A_{0}+A*e^{-\frac{t}{\lambda }}$$

(3) $$t_{1/2}=\ln\left(2\right)*\lambda$$

### Scanning electron microscopy

The success of the enzymatic treatment and intactness of the PET fibres was assessed using scanning electron microscopy (SEM). Treated and untreated samples were mounted on adhesive tape and coated with gold in argon atmosphere with a COXEM SPT-20 (COXEM, Daejeon, South Korea). The SEM images were obtained in a COXEM EM-30-plus (COXEM) using an acceleration voltage of 12–20 kV.

### Elastane separation process via non-hazardous solvent extraction

After successful removal of wool fibres by enzymatic treatment, the residual PET/EL fabric was treated with the non-hazardous organic solvent DMSO according to (Boschmeier et al., 2023b). Herein, DMSO was chosen as the favoured solvent because it is not listed under substances of very high concern in REACH. In this method, elastane is gently removed with DMSO at 120°C with a treatment duration of only 10 minutes. Full dissolution of elastane fibres was achieved by adding 1 g of textile substrate per 150 mL DMSO. Such a short treatment time leaves the obtained PET intact, the pure polymer can be removed from the reaction flask and the contaminated solvent is purified and recovered through filtration, centrifugation and distillation.

### Tensile tests on PET fibres

A PET reference was treated for 8 and 48 hours under enzymatic conditions with enzyme A and without (blank) as stated in section ‘Enzymes, characterization and treatment of wool fibres’. The same PET reference was treated for 10 minutes and 2 hours in DMSO according to the elastane separation process (ESP; see section ‘Elastane separation process via non-hazardous solvent extraction’). All treated samples were then washed in tap water and dried at 60°C for 12 hours. Possible differences in the tensile properties between the treated and reference samples were investigated by tensile tests using Vibrodyn 400 and Vibroskop 400 (Lenzing Instruments, Gampern, Austria). According to ISO 5079:2020 standard, the gauge length was set to 20 mm and a test speed of 20 mm minute^−1^ was used with a pretension weight of 100 mg. For every sample, 10 measurements were performed and changes in elongation at break, tenacity and fibre titre induced by short and long-time exposure were investigated by analysis of variance (ANOVA) with a significance level of 0.05. Before execution of the ANOVA, the standard deviations (SD) σ within a group of samples have been checked according to the criterion σ_max_ ⩽ 2σ_min_ to meet the analysis precision. The *p*-values lower than 0.05 are considered as statistically significant and a post hoc test (Tukey) was conducted to identify statistical differences between the reference and short-time treated (8 hours enzyme A, 10 minutes ESP) samples. In the Tukey-test, the absolute values of the difference in the sample means (*d*) higher than the critical value (CV) are considered as statistically significant.

## Results and discussion

### Enzyme activity determination

**Figure 1.**
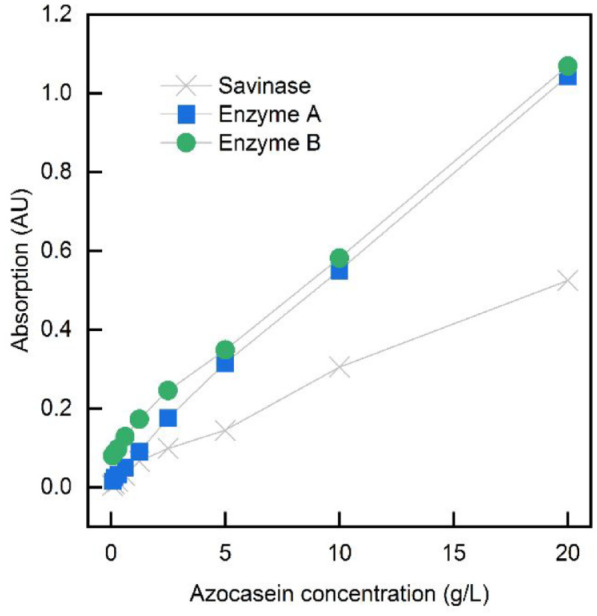
Protease activity of enzymes A, B and Savinase in dependence on the azocasein concentration.

The acceleration of the enzymatic treatment duration is crucial, since previous works reported a treatment time of 16–48 hours as described in the introduction. Therefore, two novel enzymes (enzyme A and B) were tested for the enzymatic treatment with the desire of increased activity compared to the one used in previous works. Those novel enzymes are different from already available Savinase, which belongs to the group of subtilisin proteases and can be isolated from *Bacillus lentus*. For a first performance screening, the activity assays of the three enzymes were conducted. Figure 1 shows the absorption by increasing azocasein concentrations. The two novel enzymes A and B reveal higher activities, 56.3 and 51.9 U mL^−1^, respectively, in contrast to Savinase with 29.4 U mL^−1^, which suggests an accelerated hydrolysis performance. For this reason and because Savinase seems not to be appropriate for the used substrate, the enzymatic treatment trials proceeded using enzymes A and B.

### Kinetic studies of wool removal

**Figure 2.**
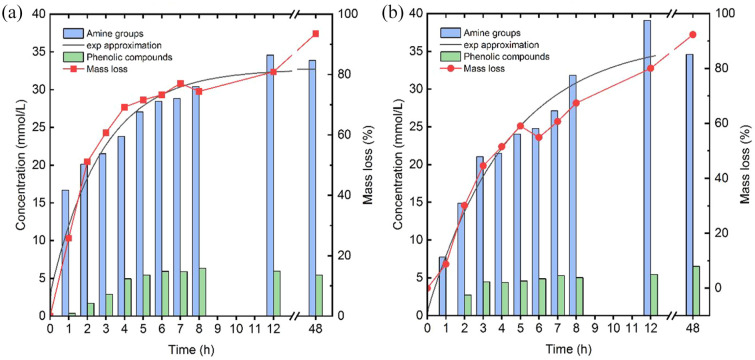
Evolution of released amino acids for (a) enzyme A and (b) enzyme B based on the detected amine and phenolic groups. The wool mass yields are obtained from the wool/PET/EL blend before post-treatment. The exponential approximation is adapted on the released amine groups. PET: polyethylene terephthalate; EL: elastane.

The hydrolysis of wool in wool/PET/EL blends by the enzymes A and B was studied in detail for timepoints 1–8, 12 and 48 hours. Mass yields were obtained from the dry samples and the hydrolysates were analysed through the Ninhydrin assay and Folin–Ciocalteu assay. Since both assays reflect the amount of wool depolymerized into amino acids, the enzyme performance can be determined. Besides a lower SD (<2.3 mmol L^−1^) and higher detected amounts compared to the Folin–Ciocalteu assay, only the Ninhydrin results are considered for the exponential approximation. Figure 2 shows the reaction progress of enzymes A and B according to the mass differences, the released amine groups and released phenolic compounds and in Table 1 the results of the exponential function approximation are displayed.

**Table 1.** Exponential function parameters for the two novel enzymes obtained from the Ninhydrin assay.

Enzyme	Parameter	Value	SD	Unit
A	A_0_	32.7	1.6	mmol L^−1^
	A	−29.8	2.5	mmol L^−1^
	λ	2.8	0.5	Hours
B	A_0_	36.6	2.1	mmol L^−1^
	A	−36.1	2.6	mmol L^−1^
	λ	4.4	0.7	Hours

SD: standard deviation.

**Figure 3.**
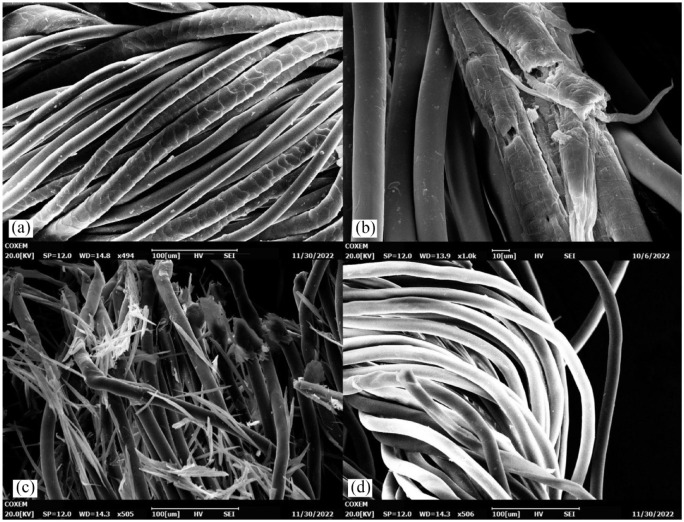
SEM images of (a) untreated wool/PET/EL sample, (b) damaged wool fibre after 1 hour enzymatic treatment and (c) spikes (residual cortical cells) visible on the PET/EL fabric directly after wool hydrolysis at 8 hours treatment time and (d) remaining PET/EL fabric after complete wool removal. SEM: scanning electron microscopy; PET: polyethylene terephthalate; EL: elastane.

For enzyme A (Figure 2(a)), half of the amine groups were detected already after *t*_1/2_ = 1.9 hours (see equation (3)), whereas a stagnation of released molecules was seen at 8 hours reaction time. Between a reaction time of 12–48 hours, only slight differences occurred. Those two circumstances might be an indication for a full wool removal after 8–12 hours, however, SEM investigation is essential to show evidence. A slower release of amine groups was detected for enzyme B (Figure 2(b)). Half of the enzyme velocity was reached only after *t*_1/2_ = 3.1 hours, which is much slower compared to enzyme A. A stagnation of maximum conversion can be expected somewhere after 8 hours. In contrast to the released amount of amine groups, the release of phenolic compounds by enzyme B was slightly delayed when compared to enzyme A. This could be due to a different cleavage pattern of the two proteases releasing more or less tyrosine containing peptides or a possible influence of released dyes. A mass loss of 50% with enzyme B revealed a necessary treatment duration of 4 hours, which is again two times longer than for enzyme A with 2 hours. By following the mass yield of the two enzymes, there are some interesting points. Firstly, it seems that removal of visible spikes, which are identified as residual cortical cells, was not complete as proved by SEM analysis (Figure 3(c)). This cortical cells make around 90% of the wool fibre mass and determine the physical and chemical properties (Giteru et al., 2023; Russell et al., 2016) and released during the enzyme degradation. Hence, the mass yields do not reach 100% yield and stagnate at around 80%. Secondly, an unexpectedly strong increase at 48 hours up to 90% was detected. Investigations revealed that with a longer lasting treatment duration the residues lost their adhesion to the fabrics and migrate in the hydrolysate, which results in a higher yield. Nevertheless, also after 48 hours those spikes can still be found in the fabric, making an adequate post-treatment essential. There is no evidence of a possible influence of the dye used in the wool fibre.

### Investigation of enzymatic wool removal coupled with necessary post-treatment

SEM was performed as a qualitative assessment method to study the wool/PET/EL textile waste morphology and support the kinetical investigation. Synthetic and wool fibres can be distinguished due to the distinct shed-like surface structure of the cuticula of wool fibres, as shown in Figure 3(a). The fibre diameter in the sample is approximately 30 µm for wool and 20 µm for synthetic ones. A distinction between elastane and PET fibres via SEM is not possible as both synthetic fibres have similar fibre widths as well as a plain surface structure. The temporal disintegration of the wool fibre was visualized via SEM analysis. Figure 3(b) shows a wool fibre after 1 hour hydrolysis treatment, resulting in a brittle fracture through the fibre and protruding tips. In agreement with earlier findings (Eslahi et al., 2013; Navone et al., 2020; Quartinello et al., 2018), the presence of sodium bisulphite as a reducing agent and SDS as surfactant enhanced the reaction rate significantly. Essentially, sodium bisulphite breaks down the robust disulphide linkages between two cysteine molecules, whereas SDS stabilizes the protein structure by forming ionic bonds with protonated-NH_4_^+^ residues (Lebedytė and Sun, 2022). This leads to the reduction of the number of intermolecular cross-linkages (Pan et al., 2016) and hence to enhanced access of the enzyme to the substrate. The cortical cells contain microfibrils which are held together by the keratin interfilamentous matrix stabilized by highly crosslinked disulphide bridges in the keratin-associated proteins (KAP) (Gong et al., 2016). When a full wool degradation is achieved (Figure 3(c)), only residual cortical cells in form of spikes can be observed within the textile fabrics. An appropriate post-treatment is necessary to fully remove those visible residues (Figure 3(c)) in order to receive a PET/EL fabric without any residual contamination. Methods were applied using tap water, ethanol and DMSO (Supplemental Table S1). This step is crucial because the presence of any contaminants in the recovered PET are undesirable. It was found that treating the hydrolysed textile samples at 60°C for 30 minutes in DMSO was the best method for fully removing the cortical cell residues as visible in Figure 3(d). The remaining PET/EL fabric stays intact during the enzymatic and DMSO treatment since no visible changes in the SEM occur, as underlined by previous literature (Boschmeier et al., 2023b; Navone et al., 2020; Quartinello et al., 2018). A full wool removal (100%) is presented here after 8.5 hours (8 hours enzyme treatment + 30 minutes DMSO cleaning), which is faster and more efficient compared to previous reported treatment durations of 24 hours (Eslahi et al., 2013), 16 hours (Navone et al., 2020) and 48 hours (Quartinello et al., 2018) and encourages the method presented in this work.

### Characterization of the wool by HPLC and SDS-PAGE

HPLC was performed to deepen the understanding of the hydrolysis reaction catalysed by enzyme A and to characterize the wool hydrolysate obtained with special regard to the potential valorization of the resulting amino acids. In Supplemental Table S2 the relative distribution of the amino acids present in the keratin hydrolysate after 8 hours reaction time is summarized.

**Figure 4.**
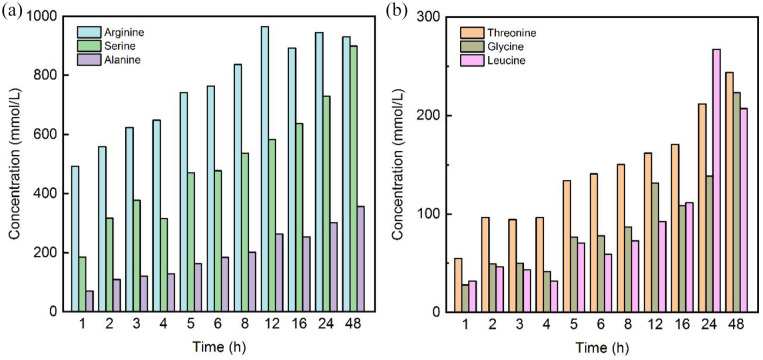
Detected amino acids in the hydrolysates of enzyme A at the chosen time stamps of (a) arginine, serine and alanine and (b) threonine, glycine and leucine.

HPLC analysis (Figure 4) showed that 60% of the wool hydrolysate consisted of arginine and serine. Mathematical approximation reveals a necessary time duration to reach 50% conversion rate (see equation (3)) with *t*_1/2_ = 1.9 hours and 6 hours for the two amino acids, respectively. If alanine (*t*_1/2_ = 6.5 hours) and threonine (*t*_1/2_ = 5.6 hours) are also considered, those four amino acids make up more than 75% of all amino acids, which has been expected (Qiu et al., 2020). Cystine, phenylalanine or aspartic acid were not detected. Noteworthy, the used HPLC-method has limitation in the analysis of cystine. On the one side, cystine is only detected as a cystine dimer, whereas on the other side the fluorescence signal of the cystine derivative is generally weak (Rawat and Maupin-Furlow, 2020).

SDS-PAGE analysis was performed to follow the progression of the cleavage of wool proteins into low-molecular (oligo-)peptides (see Supplemental Figure S1). Relative to increasing reaction times, a shift, and an increase of the bands towards low molecular mass (<10 kDa) can be seen, which indicates the increased rate of wool degradation. Molecular mass of 26.7 kDa for enzyme A and 27.3 kDa for enzyme B are traceable in this plot. Pure extracted wool exhibited fractions with molecular mass ranging from 10 to 25 kDa which correspond with molecular mass associated with the cysteine-rich KAP (Gong et al., 2016).

### Elastane separation to obtain pure PET

**Figure 5.**
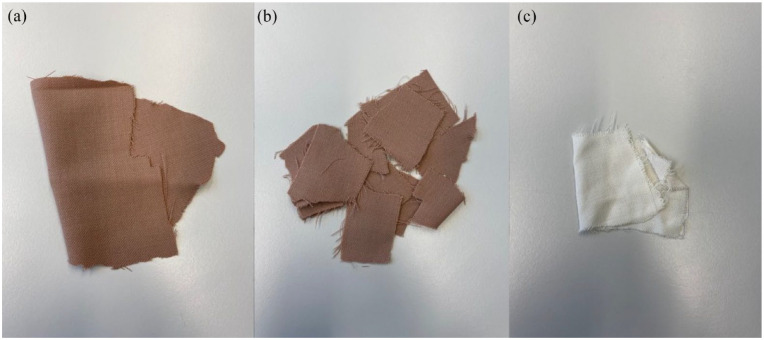
Different treatment steps of (a) the untreated wool/PET/elastane textile, (b) PET/EL fabric right after enzymatic removal of wool fibres and (c) pure PET after elastane separation. PET: polyethylene terephthalate; EL: elastane.

For further recycling steps such as melt-extrusion, a pure PET feed is essential. Therefore, the full separation of all components present in the wool/PET/EL textile is required (Figure 5(a)). Right after the accelerated enzymatic treatment, the remaining PET/EL fabric (Figure 5(b)) was treated with an organic solvent in order to remove the elastane share. Therefore, the previously introduced ESP by (Boschmeier et al., 2023b) was used. Considering the above presented removal of wool residues by DMSO washing at 60°C, wool fibre residues and elastane fibres could simultaneously be removed without damaging the PET fibres (Figure 5(c)). In Figure 5(c) is also the well-known and appreciated side effect of decolourization noticeable, which is induced by the ESP (Boschmeier et al., 2023b). A full solvent recovery was possible by filtration, centrifugation and distillation. Furthermore, the elastane share was determined by differential scanning calorimetry measurements according to the elastane quantification tool previously developed (Boschmeier et al., 2023a). With this method it is possible to accurately quantify the share of elastane in a textile product, on this substrate it was identified to be 2%. Together with the mass yields of the wool depolymerization experiments, the textile composition was calculated to be 41% wool, 57% PET and 2% elastane per mass. Initially, the compositions from the labels were used, making the calculations incorrect; however with the new textile composition, it was possible to calculate the full separation of wool and elastane fibres. There is no evidence on mass changes retrieved from the decolorization effect.

After adaption of the textile composition in percentage per mass, quick studies on the optimum enzyme concentration were conducted. Enzyme concentrations were varied (1.5, 2.5 and 5% per volume) and tests were performed in triplicate with a treatment duration of 8 hours inclusively the DMSO post-treatment. A full conversion was achieved with enzyme A with the proposed 2.5 vol.-% enzyme dispersion, showing that this volume is sufficient. All the results are shown in Supplemental Figure S2.

### Tensile properties of PET fibres after enzymatic and ESP treatment

**Figure 6.**
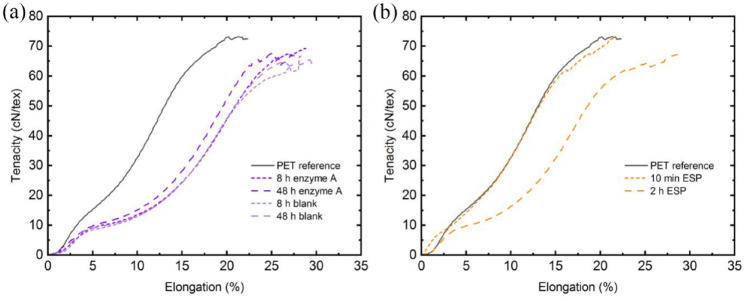
Stress–strain curves before and after treatment under (a) enzymatic and (b) ESP conditions. ESP: elastane separation process.

A first look on the stress–strain curves plotted in Figure 6 reveal a difference in the PET fibre elongations, which is underlined by ANOVA and Tukey-test results (Supplemental Table S3). Long-time exposure of 48 hours to the enzymatic conditions and 2 hours to the ESP increase the elongation significantly: all *p*-values are lower than 0.001. Additionally, a Tukey test between the untreated PET reference and 8 hours exposure with (enzyme A) and without (blank) the enzyme confirms those results (Supplemental Table S3). However, 10 minutes ESP exposure is not affecting the PET fibre elongation significantly according to the Tukey test: *d* = 1.05 < CV = 2.35. Taking the tenacity results into consideration, a tenacity reduction induced by the enzymatic conditions can be observed in Figure 6(a). The ANOVA result of the blank (*p* < 0.001) is different to the ANOVA result of the enzyme (*p* = 0.07) and shows an enzyme-catalysed statistically significant influence on the PET fibre tenacity. Furthermore, Tukey test confirms a significant impact on the tenacity in both variants: with and without enzyme A. It can be concluded that the enzymatic conditions have an impact on the PET fibre tenacity. Especially the amorphous regions in the PET are susceptible to alkali treatment (Chen et al., 2015). Additionally, the temperature of 50°C and long exposure (8 hours) can influence the molecular chain orientation, which is responsible for the mechanical properties (Qin et al., 2018). However, a direct comparison of the average tenacities reveals a reduction of only 5% (PET reference: 70.8 cN/tex; 8 hours Enzyme A: 67.5 cN/tex). In contrast to the enzymatic treatment, no influence on the tenacity induced by ESP treatment (Figure 6(b)) is observed. That noteworthy outcome is underlined by the ANOVA (*p* = 0.01) and Tukey-test results and highlights the ESP. A fibre titre swelling could be observed by ANOVA only for the enzyme A samples (*p* = 0.31), but Tukey test reveals no significant difference.

### Field test of the novel enzyme A (NS 59161) on post-consumer textile waste

Based on the successful demonstration of an optimized enzymatic removal of wool, first upscaling trials were carried out on five post-consumer textile waste samples containing PET/wool in unknown shares. For this purpose, an incubator with a reaction volume of 4 L was used and the optimal parameters from the wool/PET/EL sample were applied on the PET/wool samples: 8-hour enzymatic treatment coupled with 30 minutes washing in DMSO. The applicability of the developed methods is still far from semi-industrial scale, nevertheless the results are of great interest for the textile recycling sector as 61 g of recovered PET could be generated from 200 g PET/wool feed. It was possible to increase the ratio between substrate and reaction volume from 3% to 5% (w/v) and a pure PET fabric was obtained. The post-treatment enabled the removal of cortical cell residues and contaminants adhered to the fabric surface. PET purity was confirmed by SEM (Supplemental Figure S3), which showed no indication of any wool fibre residues on four of five samples (Supplemental Figures S4–S8). Only one of the samples (Supplemental Figure S6) contained little amounts of undamaged wool fibres which could be ascribed to the high fibre density caused by tight weaving (Supplemental Table S4). For this dense fabric type, the accessibility of the enzyme to the wool fibre is hindered. To overcome this obstacle, shredding of feed material leads to more surface area which enhances the enzymes accessibility to the wool fibres (Gritsch et al., 2023). Fashion fabric related details such as grammage, fibre titre, yarn titre, yarn twists, etc. exhibiting then low relevance to the process. Since recovered PET needs to be shredded for thermo-mechanical recycling anyway, such pre-processing does not influence further steps.

## Conclusion

In this work, a new approach for recycling of textile waste consisting of wool/PET/EL is presented, making such a textile composition first-time recyclable. Wool fibres were successfully degraded with two novel enzymes into its amino acids. From the kinetical investigation, NS 59161 (enzyme A) was chosen as the best performer over NS 29083 (enzyme B). According to the conducted assays, a treatment duration of 8 hours is sufficient for full wool conversion and even a double acceleration compared to the latest published treatment duration of 16 hours. A quick cleaning step of 30 minutes with the non-hazardous solvent DMSO removes enzymatic treatment residues. Elastane fibres were successfully removed through the ESP in order to obtain pure PET. Excessive solvent was recovered via distillation allowing to ameliorate the overall material consumption and lowering the generation of waste for a future sustainable process. Furthermore, a full solvent recovery is essential on large scale the latest to lower process costs. Tensile measurements on the PET fibres reveal no changes in the polymer matrix induced by the ESP, but slight changes through the enzymatic treatment. Since it is common practice to blend recycled PET fibres with virgin PET fibres for new textile yarn production, the small changes are negligible. Finally, a field test on five pre-sorted post-consumer textile waste samples consisting of wool/PET proved the novel enzyme. In four out of five textile waste samples the wool share could successfully be removed and pure PET was visualized by SEM imaging. Only in one very densely woven sample some wool fibres were left, this minor issue could be tackled by milling the samples beforehand. These results can be applied on any wool and/or EL containing textile waste since both processes are based on methods that are highly selective and any other fibre type is not affected.

A potential upscaling of enzymatic treatment of such textile waste samples seems promising, because washing with a detergent, centrifugation and a temperature control is the same principle as in a washing machine. One drawback is the potential denaturation of the enzymes, which prevents a circular use of the enzymes within the process (Navone et al., 2020).

Current recycling processes in the textile sector primarily require pure input streams, such as cellulose used on an industrial scale for producing high-quality lyocell fibres (Haule et al., 2016; Liu et al., 2019). Although literature describes processes, albeit only on a laboratory scale, where textiles consisting of two polymers can be recycled after separating one component, particularly PET extracted from cotton (Piribauer et al., 2021). Textiles composed of more than two polymers have not been recyclable so far. With the method presented here, it is now possible to prepare even complex textile waste for recycling processes. This study demonstrates for the first time that textiles composed of three fibre types can also be made recyclable. In principle, this method can be applied to other material combinations as well. We are convinced that this represents an important, albeit not the final, step for the textile industry towards a circular economy.

## Supplemental Material

sj-pdf-1-wmr-10.1177_0734242X241276089 – Supplemental material for Recovery of pure PET from wool/PET/elastane textile waste through step-wise enzymatic and chemical processing Supplemental material, sj-pdf-1-wmr-10.1177_0734242X241276089 for Recovery of pure PET from wool/PET/elastane textile waste through step-wise enzymatic and chemical processing by Emanuel Boschmeier, Daniella Mehanni, Viktor Laurin Sedlmayr, Yury Vetyukov, Sophia Mihalyi, Felice Quartinello, Georg M Guebitz and Andreas Bartl in Waste Management & Research

## References

[bibr1-0734242X241276089] Aronsson J Persson A (2020) Tearing of post-consumer cotton T-shirts and jeans of varying degree of wear. Journal of Engineered Fibers and Fabrics 15: 155892502090132.

[bibr2-0734242X241276089] Bianco I Gerboni R Picerno G (2022) Life cycle assessment (LCA) of MWool^®^ recycled wool fibers. Resources 11: 41.

[bibr3-0734242X241276089] Boschmeier E Archodoulaki V-M Schwaighofer A , et al. (2023a). A novel quantification tool for elastane in textiles using thermal treatment. Polymer Testing 118: 107920.

[bibr4-0734242X241276089] Boschmeier E Archodoulaki V-M Schwaighofer A , et al. (2023b). New separation process for elastane from polyester/elastane and polyamide/elastane textile waste. Resources, Conservation and Recycling 198: 107215.

[bibr5-0734242X241276089] Boschmeier E Ipsmiller W Bartl A (2024) Market assessment to improve fibre recycling within the EU textile sector. Waste Management & Research 42: 135–145. 37313862 10.1177/0734242X231178222 PMC10832323

[bibr6-0734242X241276089] Chen T Zhang W Zhang J (2015) Alkali resistance of poly(ethylene terephthalate) (PET) and poly(ethylene glycol-co-1,4-cyclohexanedimethanol terephthalate) (PETG) copolyesters: The role of composition. Polymer Degradation and Stability 120: 232–243.

[bibr7-0734242X241276089] Deopura BL Alagirusamy R ; Textile Institute (Eds.) (2008) Polyesters and polyamides. Boca Raton, FL: CRC Press.

[bibr8-0734242X241276089] EC (2018) Directive (EU) 2018/851 of the European Parliament and of the Council of 30 May 2018 amending Directive 2008/98/EC on waste. Official Journal of the European Union.

[bibr9-0734242X241276089] EC (2022) Communication from the Commission to the European Parliament, the Council, the European Economic and Social Committee and the Committee of the Regions EU Strategy for Sustainable and Circular Textiles, COM (2022) 141 final.

[bibr10-0734242X241276089] EC (2023) Proposal for a Directive of the European Parliament and of the Council amending Directive 2008/98/EC on waste COM/2023/420. European Commission.

[bibr11-0734242X241276089] EC (2024) European Parliament legislative resolution of 23 April 2024 on the proposal for a regulation of the European Parliament and of the Council establishing a framework for setting eco-design requirements for sustainable products and repealing Directive 2009/125/EC (COM(2022)0142–C9-0132/2022–2022/0095(COD)).

[bibr12-0734242X241276089] El-Sayed H (2022) The current status and future insight into the production of machine-washable wool. Journal of Natural Fibers 19: 10293–10305.

[bibr13-0734242X241276089] Eslahi N Dadashian F Nejad NH (2013) An investigation on keratin extraction from wool and feather waste by enzymatic hydrolysis. Preparative Biochemistry and Biotechnology 43: 624–648. 23768110 10.1080/10826068.2013.763826

[bibr14-0734242X241276089] Fang Z Zhang J Liu B , et al. (2013) Biodegradation of wool waste and keratinase production in scale-up fermenter with different strategies by *Stenotrophomonas maltophilia* BBE11-1. Bioresource Technology 140: 286–291. 23708787 10.1016/j.biortech.2013.04.091

[bibr15-0734242X241276089] Giteru SG Ramsey DH Hou Y , et al. (2023) Wool keratin as a novel alternative protein: A comprehensive review of extraction, purification, nutrition, safety, and food applications. Comprehensive Reviews in Food Science and Food Safety 22: 643–687. 36527315 10.1111/1541-4337.13087

[bibr16-0734242X241276089] Gong H Zhou H Forrest R , et al. (2016) Wool keratin-associated protein genes in sheep – A review. Genes (Basel) 7: 24. 27240405 10.3390/genes7060024 PMC4929423

[bibr17-0734242X241276089] Gritsch SM Mihalyi S Bartl A , et al. (2023) Closing the cycle: Enzymatic recovery of high purity glucose and polyester from textile blends. Resources, Conservation and Recycling 188: 106701.

[bibr18-0734242X241276089] Haule LV Carr CM Rigout M (2016) Preparation and physical properties of regenerated cellulose fibres from cotton waste garments. Journal of Cleaner Production 112: 4445–4451.

[bibr19-0734242X241276089] Ipsmiller W Bartl A (2022) Sourcing and re-sourcing end-of-use textiles. In: Weis JS De Falco F Cocca M (eds.) Polluting textiles, 1st ed. London, New York: Routledge, pp.214–244.

[bibr20-0734242X241276089] Lang WT Mehta SA Thomas MM , et al. (2023) Chemical recycling of polyethylene terephthalate, an industrial and sustainable opportunity for Northwest of England. Journal of Environmental Chemical Engineering 11: 110585.

[bibr21-0734242X241276089] Lebedytė M Sun D (2022) A review: Can waste wool keratin be regenerated as a novel textile fibre via the reduction method? The Journal of the Textile Institute 113: 1750–1766.

[bibr22-0734242X241276089] Li Q (2022) Perspectives on converting keratin-containing wastes into biofertilizers for sustainable agriculture. Frontiers in Microbiology 13: 918262. 35794912 10.3389/fmicb.2022.918262 PMC9251476

[bibr23-0734242X241276089] Lindström K Sjöblom T Persson A , et al. (2020) Improving mechanical textile recycling by lubricant pre-treatment to mitigate length loss of fibers. Sustainability 12: 8706.

[bibr24-0734242X241276089] Liu W Liu S Liu T , et al. (2019) Eco-friendly post-consumer cotton waste recycling for regenerated cellulose fibers. Carbohydrate Polymers 206: 141–148. 30553307 10.1016/j.carbpol.2018.10.046

[bibr25-0734242X241276089] Mokrejs P Svoboda P Hrncirik J , et al. (2011) Processing poultry feathers into keratin hydrolysate through alkaline-enzymatic hydrolysis. Waste Management & Research 29: 260–267. 20483878 10.1177/0734242X10370378

[bibr26-0734242X241276089] Navone L Moffitt K Hansen K-A , et al. (2020) Closing the textile loop: Enzymatic fibre separation and recycling of wool/polyester fabric blends. Waste Management 102: 149–160. 31678801 10.1016/j.wasman.2019.10.026

[bibr27-0734242X241276089] Pan F Lu Z Tucker I , et al. (2016) Surface active complexes formed between keratin polypeptides and ionic surfactants. Journal of Colloid and Interface Science 484: 125–134. 27599381 10.1016/j.jcis.2016.08.082

[bibr28-0734242X241276089] Piribauer B Bartl A Ipsmiller W (2021) Enzymatic textile recycling – Best practices and outlook. Waste Management & Research 39: 1277–1290. 34238113 10.1177/0734242X211029167

[bibr29-0734242X241276089] Poon JPH Peng P Atkinson JD (2024) Industrial and textile waste trade: Multilayer network and environmental policy effects. Waste Management 177: 146–157. 38325015 10.1016/j.wasman.2024.01.048

[bibr30-0734242X241276089] Qin Y Qu M Kaschta J , et al. (2018) Comparing recycled and virgin poly (ethylene terephthalate) melt-spun fibres. Polymer Testing 72: 364–371.

[bibr31-0734242X241276089] Qiu J Wilkens C Barrett K , et al. (2020) Microbial enzymes catalyzing keratin degradation: Classification, structure, function. Biotechnology Advances 44: 107607. 32768519 10.1016/j.biotechadv.2020.107607 PMC7405893

[bibr32-0734242X241276089] Quartinello F Vajnhandl S Volmajer Valh J , et al. (2017) Synergistic chemo-enzymatic hydrolysis of poly(ethylene terephthalate) from textile waste. Microbial Biotechnology 10: 1376–1383. 28574165 10.1111/1751-7915.12734 PMC5658601

[bibr33-0734242X241276089] Quartinello F Vecchiato S Weinberger S , et al. (2018) Highly selective enzymatic recovery of building blocks from wool-cotton-polyester textile waste blends. Polymers (Basel) 10: 1107. 30961032 10.3390/polym10101107 PMC6403871

[bibr34-0734242X241276089] Rawat M Maupin-Furlow JA (2020) Redox and thiols in Archaea. Antioxidants (Basel) 9: 381. 32380716 10.3390/antiox9050381 PMC7278568

[bibr35-0734242X241276089] Russell S Swan P Trebowicz M , et al. (2016) Review of wool recycling and reuse. In: Fangueiro R Rana S (eds.) Natural fibres: Advances in science and technology towards industrial applications, vol. 12. Dordrecht: Springer Netherlands, pp.415–428.

[bibr36-0734242X241276089] Sonnendecker C Oeser J Richter PK , et al. (2022) Low carbon footprint recycling of post-consumer PET plastic with a metagenomic polyester hydrolase. ChemSusChem 15: e202101062. 10.1002/cssc.202101062 PMC9303343 34129279

[bibr37-0734242X241276089] Testa F Nucci B Iraldo F , et al. (2017) Removing obstacles to the implementation of LCA among SMEs: A collective strategy for exploiting recycled wool. Journal of Cleaner Production 156: 923–931.

[bibr38-0734242X241276089] Textile Exchange (2022) Preferred Fiber & Materials – Market Report. Available at: https://textileexchange.org/app/uploads/2022/10/Textile-Exchange_PFMR_2022.pdf (accessed 14 February 2023).

[bibr39-0734242X241276089] Tournier V Topham CM Gilles A , et al. (2020) An engineered PET depolymerase to break down and recycle plastic bottles. Nature 580: 216–219. 32269349 10.1038/s41586-020-2149-4

[bibr40-0734242X241276089] Villa ALV Aragão MRS Dos Santos EP , et al. (2013) Feather keratin hydrolysates obtained from microbial keratinases: Effect on hair fiber. BMC Biotechnology 13: 15. 23414102 10.1186/1472-6750-13-15 PMC3621039

[bibr41-0734242X241276089] Wiedemann SG Biggs L Nebel B , et al. (2020) Environmental impacts associated with the production, use, and end-of-life of a woollen garment. The International Journal of Life Cycle Assessment 25: 1486–1499.

