# Supplementary material for: Recovery of pure PET from wool/PET/elastane textile waste through step-wise enzymatic and chemical processing
Source: Waste Manag Res. 2024 Sep 20;43(6):969–79. doi: 10.1177/0734242X241276089 (PMC12106927; doi:10.1177/0734242X241276089)
Supplement: sj-pdf-1-wmr-10.1177_0734242X241276089 – Supplemental material for Recovery of pure PET from wool/PET/elastane textile waste through step-wise enzymatic and chemical processing [file sj-pdf-1-wmr-10.1177_0734242X241276089.pdf]

Supplementary material

Recovery of pure PET from wool/PET/elastane textile waste through step-wise enzymatic and chemical processing

Authors: Emanuel Boschmeier[1], Daniella Mehanni[1], Viktor Laurin Sedlmayr[1], Yury Vetyukov[2], Sophia Mihalyi[3], Felice Quartinello[3], Georg M. Guebitz[3] and Andreas Bartl[1]

[1] Institute of Chemical, Environmental and Bioscience Engineering, TU Wien, Getreidemarkt 9/166, 1060 Vienna, Austria
[2] Institute of Mechanics and Mechatronics, TU Wien, Getreidemarkt 9/325, 1060 Vienna, Austria
[3] University of Natural Resources and Life Sciences, Vienna, Department of Agrobiotechnology, IFA-Tulln, Institute of Environmental Biotechnology, Konrad-Lorenz-Strasse 20, 3430 Tulln an der Donau, Austria

Corresponding author: andreas.bartl@tuwien.ac.at

Table S1. Washing methods applied to remove woollen fibrous residues after enzymatic treatment.

Method	Description
1	Treatment in 100 mL H_2O at 100°C for 30 minutes
2	Washing in 100 mL ethanol (96%) at 60°C for 30 minutes
3	Treatment in 100 mL DMSO at 60°C for 30 minutes

Table S2. Amino acid composition of enzyme A after a reaction time of 8 hours determined by HPLC. For the four amino acids with the highest share, the results of the exponential approximation are stated out.

Amino acid	Share	A_0	A	λ
	%	mmol L^{-1}	mmol L^{-1}	h
Arginine	36.7	911.9	-818.5	2.8
Serine	23.5	821.6	-721.5	8.8
Alanine	8.8	338.5	-309.5	9.3
Threonine	6.6	223.4	-195.4	8.1
Tyrosine	4.1			
Asparagine	3.9			
Glycine	3.8			
Leucine	3.2			
Glutamic acid	3.1			
Isoleucine	2.9			
Valine	< 1			
Histidine	< 1			
Tryptophane	< 1			
Lysine	< 1			
Glutamine	< 1			
Methionine	-			
Aspartic acid	-			
Norvaline	-			
Cystine	-			
Phenylalanine	-			

Table S3. ANOVA and Tukey-Test results obtained from the tensile measurements of PET fibres.

ANOVA with F_{crit} = 3.35

Parameter	Value	Enzyme A	Blank	DMSO
Tenacity	F	2.93	22.35	5.65
	p	0.07	<0.001	0.01
Elongation	F	47.06	83.04	25.07
	p	<0.001	<0.001	<0.001
Titer	F	1.21	7.85	5.79
	p	0.31	0.002	0.01

Tukey-Test

Parameter	Value	Enzyme A	Blank	DMSO
Tenacity	d	3.28	9.28	2.24
	CV	3.00	2.87	3.39
Elongation	d	6.98	6.64	1.05
	CV	1.54	1.35	2.35
Titer	d	0.16	0.47	0.22
	CV	0.22	0.25	0.25

Table S4. Post-consumer textile waste samples used for enzymatic wool removal. The unknown wool shares were determined by mass before and after hydrolysis inclusive post-treatment.

Sample	Mass untreated	Mass treated	Wool share calc.	Proof of full wool removal
	g	g	w.-%	
#1	3.046	2.087	31	Yes, Figure S4
#2	2.987	1.621	46	Yes, Figure S5
#3	3.028	1.931	36	No, Figure S6
#4	3.265	2.187	33	Yes, Figure S7
#5	3.197	2.529	21	Yes, Figure S8

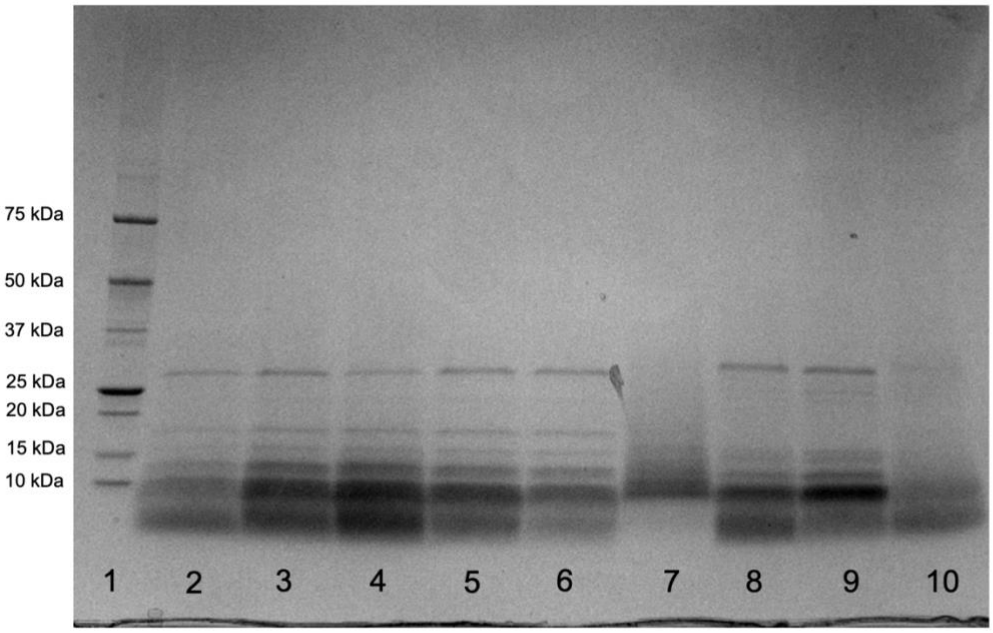

Figure S1. SDS-PAGE analysis of keratin hydrolysate fractions at given reaction times: (1) marker; (7) pure keratin extracted from untreated sample; bands associated with enzyme A: (2) 48 h, (3) 16 h, (4) 8 h, (5) 2 h, (6) 1 h; Enzyme B: (8) 8 h, (9) 16 h, (10) 48.

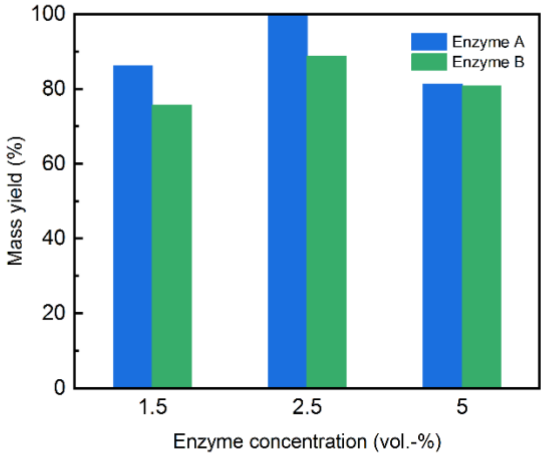

Figure S2. Variation of the enzyme concentration and post-treatment to remove cortical cell spikes with a treatment duration of 8 hours. Only with enzyme A, a full wool removal was possible with the proposed 2.5 vol.-% enzyme concentration.

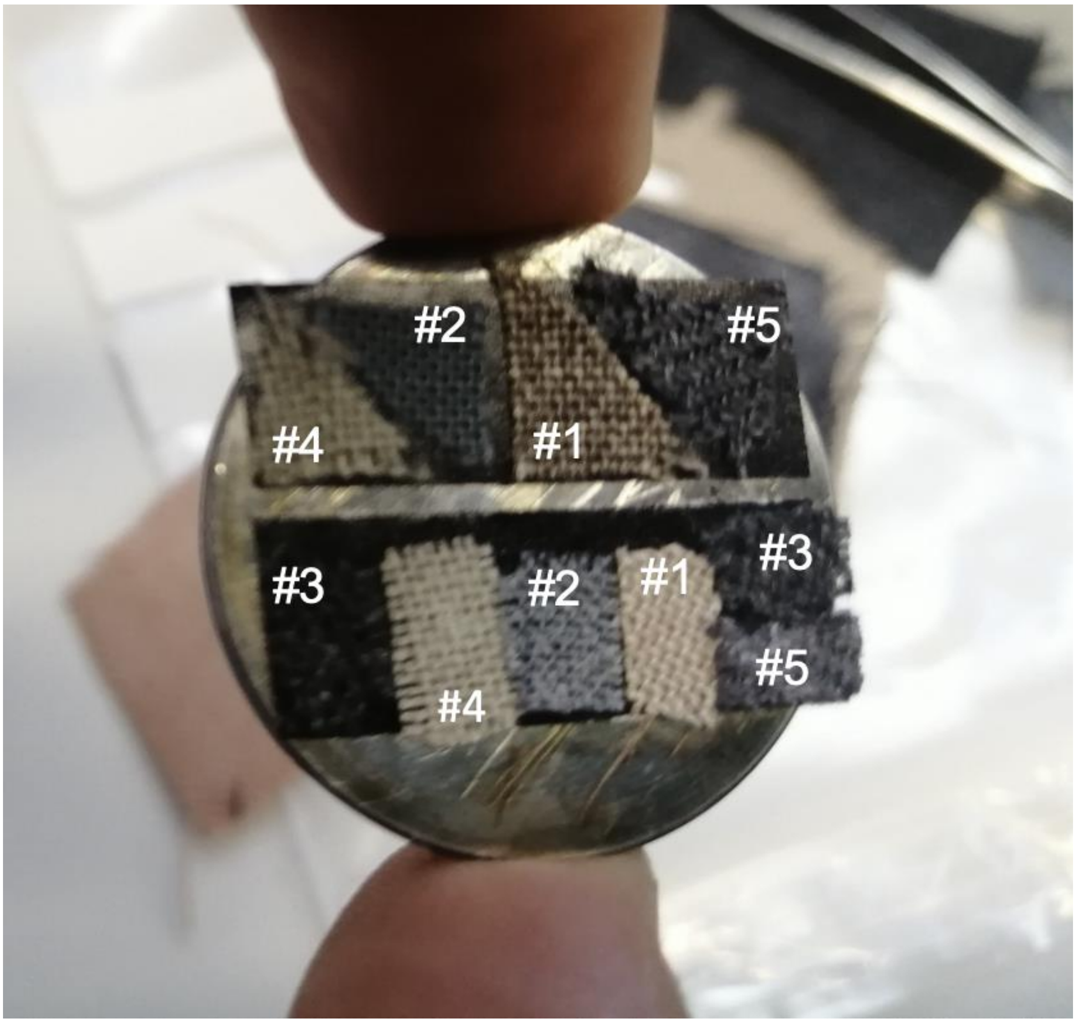

Figure S3. SEM sample holder with cut-outs of the five post-consumer PET/wool samples ready for investigation.

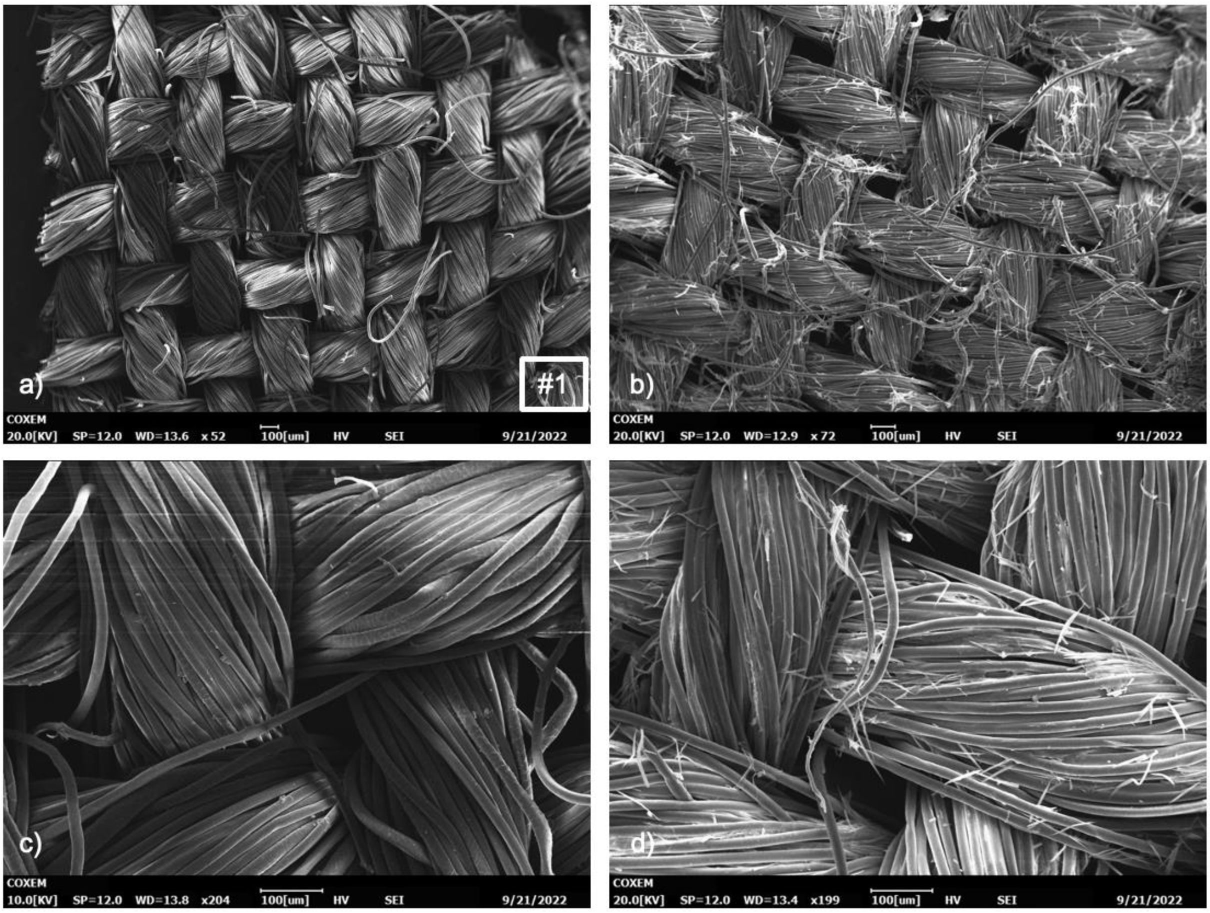

Figure S4. SEM images of post-consumer sample #1 in different resolutions of the untreated (a and c) and enzymatic treated (b and d) before the solvent post-treatment to remove residual cortical cells.

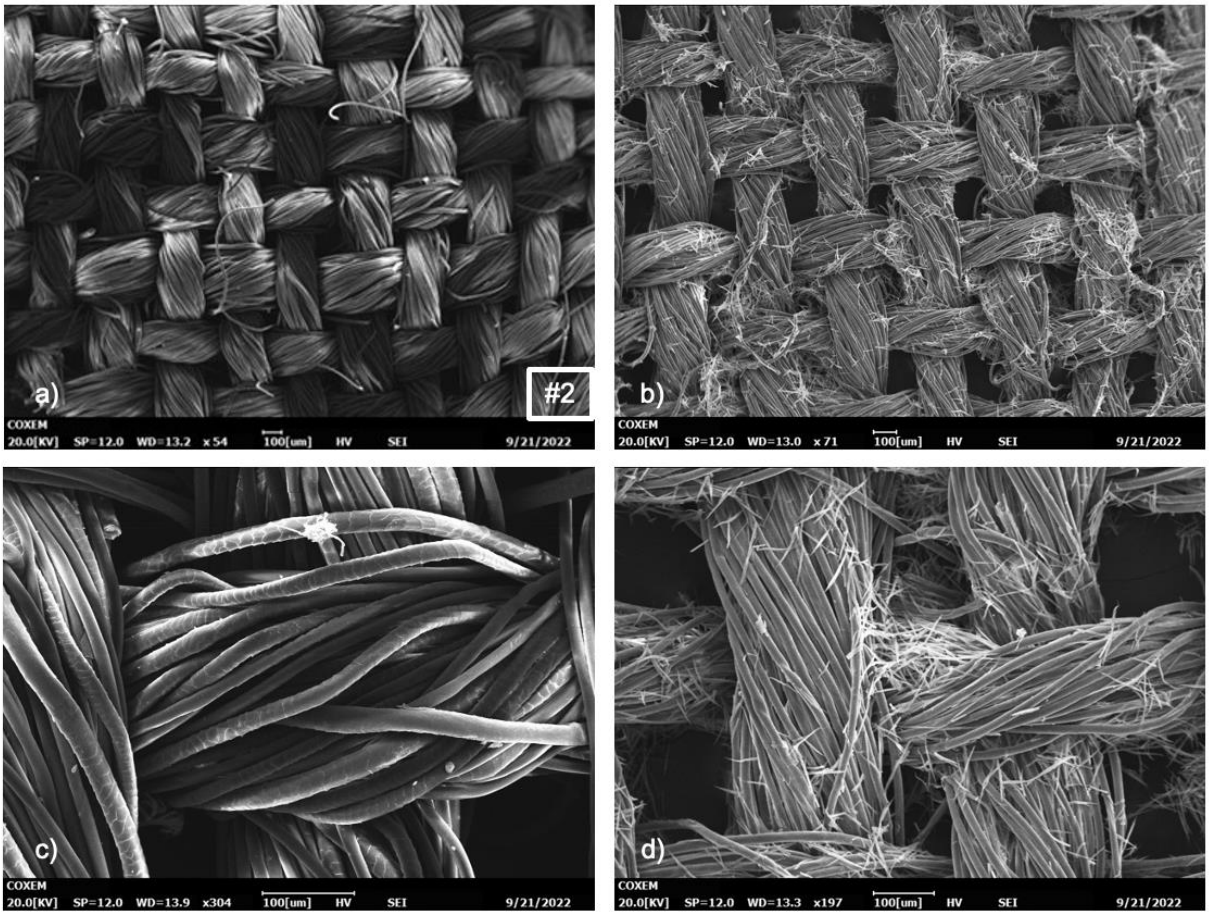

Figure S5. SEM images of post-consumer sample #2 in different resolutions of the untreated (a and c) and enzymatic treated (b and d) before the solvent post-treatment to remove residual cortical cells.

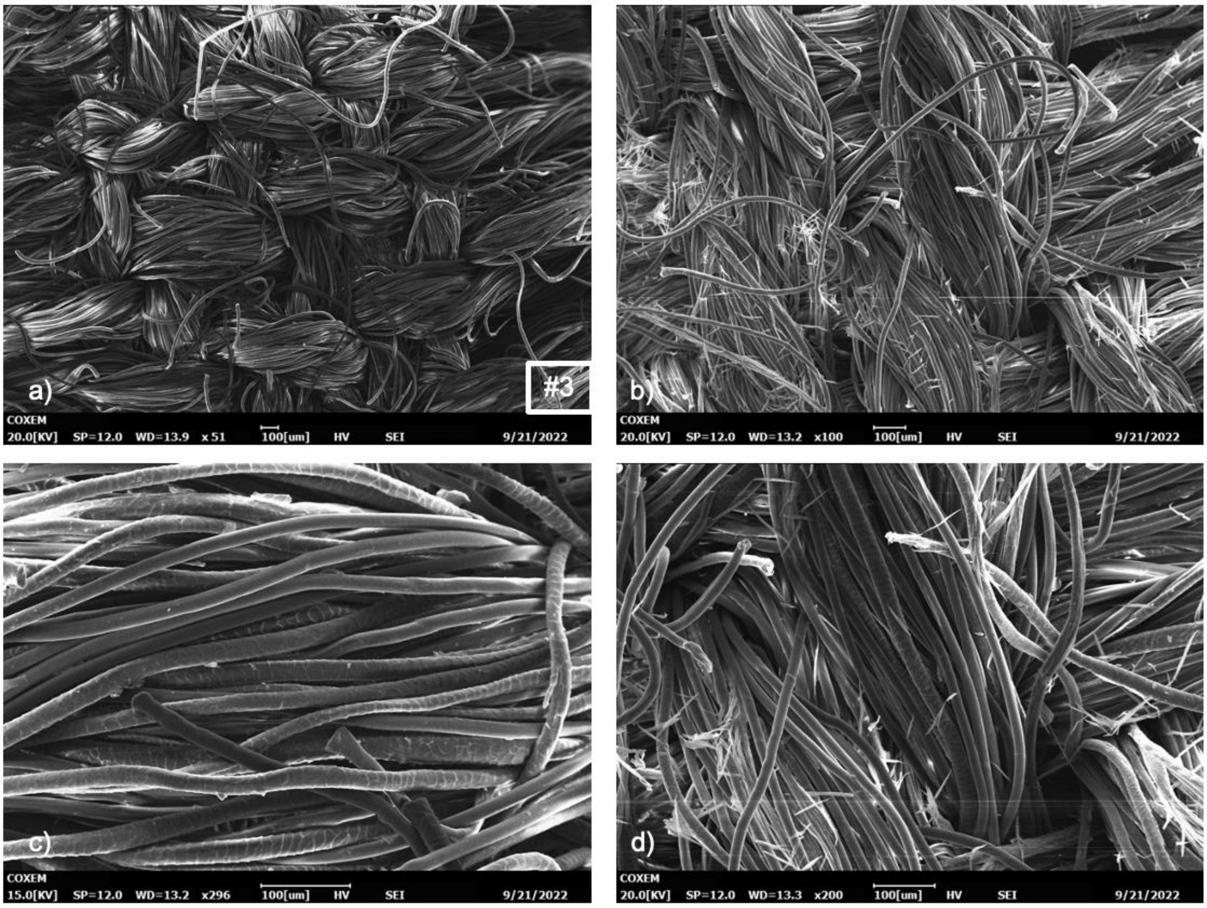

Figure S6. SEM images of post-consumer sample #3 in different resolutions of the untreated (a and c) and enzymatic treated (b and d) before the solvent post-treatment to remove residual cortical cells. Due to the tight weaving, still some wool fibres are left. However, fabric shredding will enhance the enzymes accessibility.

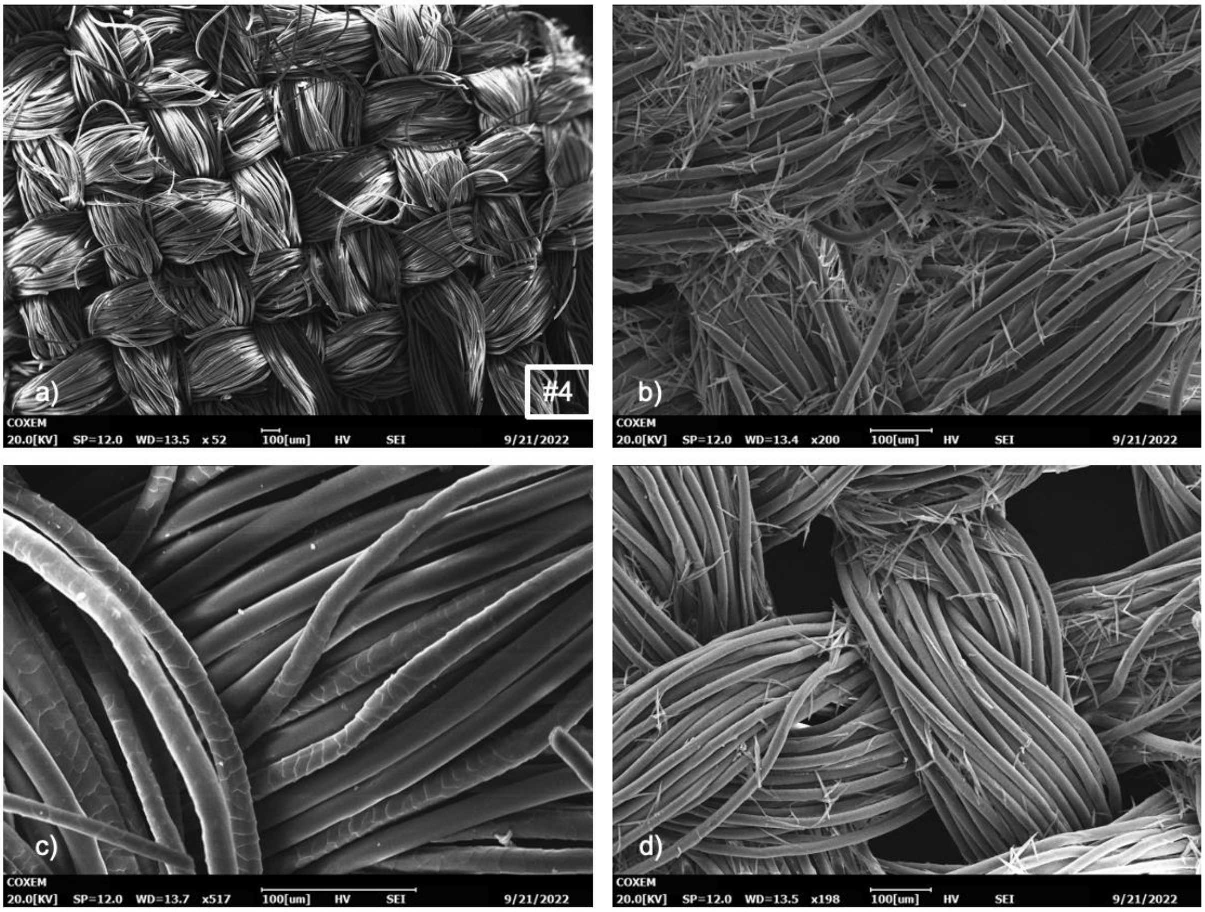

Figure S7. SEM images of post-consumer sample #4 in different resolutions of the untreated (a and c) and enzymatic treated (b and d) before the solvent post-treatment to remove residual cortical cells.

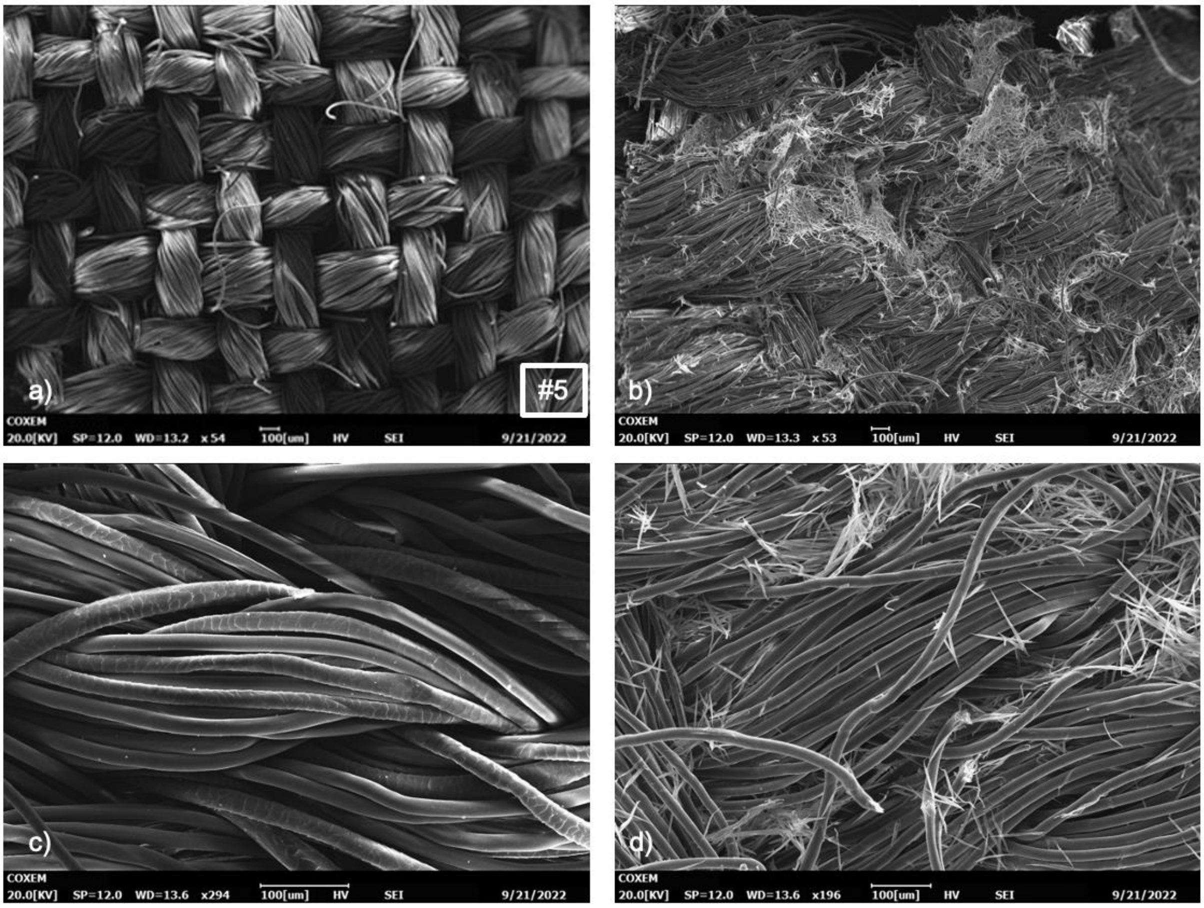

Figure S8. SEM images of post-consumer sample #5 in different resolutions of the untreated (a and c) and enzymatic treated (b and d) before the solvent post-treatment to remove residual cortical cells.
